# The Disulfide Bond Cys255-Cys279 in the Immunoglobulin-Like Domain of Anthrax Toxin Receptor 2 Is Required for Membrane Insertion of Anthrax Protective Antigen Pore

**DOI:** 10.1371/journal.pone.0130832

**Published:** 2015-06-24

**Authors:** Pedro Jacquez, Gustavo Avila, Kyle Boone, Agamyrat Altiyev, Jens Puschhof, Roland Sauter, Emma Arigi, Blanca Ruiz, Xiuli Peng, Igor Almeida, Michael Sherman, Chuan Xiao, Jianjun Sun

**Affiliations:** 1 Department of Biological Sciences & Border Biomedical Research Center, University of Texas at El Paso, 500 West University Avenue, El Paso, Texas, 79968, United States of America; 2 Department of Chemistry, University of Texas at El Paso, 500 West University Avenue, El Paso, Texas, 79968, United States of America; 3 Bioinformatics Program of University of Texas at El Paso, 500 West University Avenue, El Paso, Texas, 79968, United States of America; 4 Department of Biochemistry and Molecular Biology, Sealy Center for Structural Biology and Molecular Biophysics, University of Texas Medical Branch, Galveston, Texas, 77555, United States of America; 5 China National Key Laboratory of Agricultural Microbiology, Huazhong Agriculture University, Wuhan, 430070, P. R. China; Institute Pasteur, FRANCE

## Abstract

Anthrax toxin receptors act as molecular clamps or switches that control anthrax toxin entry, pH-dependent pore formation, and translocation of enzymatic moieties across the endosomal membranes. We previously reported that reduction of the disulfide bonds in the immunoglobulin-like (Ig) domain of the anthrax toxin receptor 2 (ANTXR2) inhibited the function of the protective antigen (PA) pore. In the present study, the disulfide linkage in the Ig domain was identified as Cys255-Cys279 and Cys230-Cys315. Specific disulfide bond deletion mutants were achieved by replacing Cys residues with Ala residues. Deletion of the disulfide bond C255-C279, but not C230-C315, inhibited the PA pore-induced release of the fluorescence dyes from the liposomes, suggesting that C255-C279 is essential for PA pore function. Furthermore, we found that deletion of C255-C279 did not affect PA prepore-to-pore conversion, but inhibited PA pore membrane insertion by trapping the PA membrane-inserting loops in proteinaceous hydrophobic pockets. Fluorescence spectra of Trp59, a residue adjacent to the PA-binding motif in von Willebrand factor A (VWA) domain of ANTXR2, showed that deletion of C255-C279 resulted in a significant conformational change on the receptor ectodomain. The disulfide deletion-induced conformational change on the VWA domain was further confirmed by single-particle 3D reconstruction of the negatively stained PA-receptor heptameric complexes. Together, the biochemical and structural data obtained in this study provides a mechanistic insight into the role of the receptor disulfide bond C255-C279 in anthrax toxin action. Manipulation of the redox states of the receptor, specifically targeting to C255-C279, may become a novel strategy to treat anthrax.

## Introduction

In order to overcome the host defense system, pathogenic bacteria deliver toxins into the cytoplasm of host cells and disrupt the key cellular metabolic pathways. Most of the intracellularly acting bacterial toxins enter into host cells through receptor-mediated endocytosis [1]. Elucidation of molecular mechanism how receptors perform their roles in toxin actions will enhance our understanding of host-pathogen interactions and facilitate development of novel therapeutics against infection.

Anthrax toxin, one of the major virulence factors of *Bacillus anthracis*, is a tripartite A-B toxin. It is composed of two catalytic moieties, edema factor (EF) and lethal factor (LF), and a receptor-binding/pore-forming moiety, protective antigen (PA) [2]. The full-length PA is an 83-kDa protein. After binding to cell-surface receptors, PA is cleaved by furin or a furin-like protease to generate an active, 63-kDa form (PA_63_) [3]. PA_63_ then oligomerizes into a heptameric or octameric receptor-bound prepore [4,5], which contains high-affinity binding sites for EF and LF [6]. The toxin-receptor complexes are internalized by receptor-mediated endocytosis, and the prepore undergoes an acidic pH-dependent conformational rearrangement within the endosome to form a cation-selective, transmembrane pore [7,8]. The PA pore mediates translocation of EF and LF across the endosomal membrane into the cytosol, where EF, an 89-kDa calmodulin-dependent adenylate cyclase, elevates levels of cAMP [9], and LF, a 90-kDa zinc protease, inactivates mitogen-activated protein kinase kinases [10].

Anthrax toxin receptors play an essential role in anthrax toxin action. They provide the toxin with a high-affinity anchor on the membranes and a path of entry into the cells. Two cellular receptors for PA have been identified: ANTXR1 (or TEM8) [11] and ANTXR2 (or CMG2) [12]. Recently, it has been shown that the lethality of anthrax toxin for mice is primarily mediated by ANTXR2 and that ANTXR1 plays only a minor role [13]. ANTXR2 is a type-I transmembrane protein and comprises an extracellular von Willebrand Factor A domain (VWA, residues 38–218), a stalk region (residues 219–318), a single-pass transmembrane domain, and a cytoplasmic domain. The VWA domain is identified as a high-affinity PA binding domain that binds to PA in a one-to-one stoichiometry [14,15]. The MIDAS (metal ion dependent association site) of the VWA domain binds to domains 4 and 2 of PA [16,17]. Moreover, the VWA domain functions as a molecular clamp or switch that prevents PA prepore-to-pore conversion at neutral pH [16,18,19]. Most recently, a study has shown that binding of the VWA domain largely increases the stability of PA and the effect reaches up to 70 Å from the receptor binding interface [20]. While the role of VWA domain in receptor-PA interaction and in PA pore formation has been well studied [21–25], little is known about the structure and function of the stalk region (219–318). Recently, we have found that the stalk region is an immunoglobulin-like (Ig) domain and the disulfide bonds in this domain are essential for functioning of the PA pore [26]. In the present study, we demonstrated that deletion of the disulfide bond C255-C279 in the Ig domain induced significant conformational changes on the receptor Ig and VWA domains, which inhibited PA pore membrane insertion by trapping the PA membrane-inserting loops in hydrophobic pockets of the unfolded receptor.

## Materials and Methods

### Gene cloning and site-directed mutagenesis

The gene encoding the mature ectodomain of ANTXR2 (residues 38–318, termed R318) was cloned into pCOLD-TF vector (Takara, Clontech Laboratories Inc.). The Cys residues on the Ig domain were replaced with Ala residues as indicated in the text by site-directed mutagenesis (Agilent), and the mutations were confirmed by DNA sequencing.

### Protein expression and purification

R318 and the C/A mutants of R318 were expressed and purified as previously described [27]. The VWA domain (residues 38–218, termed R218), the full length PA_83_, and the heptamer (PA_63_)_7_ were expressed and purified as previously described [4,15,28,29].

### Tryptophan fluorescence

5 μM of R218, R318, the C/A mutants of R318 were treated with/without 10 mM TCEP in 20 mM Tris-HCl, 100 mM NaCl, pH 7.3. Emission spectra of Trp59 fluorescence were recorded in an ISS-K2 multiphase frequency and modulation fluorometer from 300–370 nm with excitation at 290 nm. A 295 nm long-pass filter was used to reduce the background. The spectra were calibrated using the same buffers with/without TCEP.

### Pyrene fluorescence

PA_83_ carrying the mutation N306C was labeled with pyrene as previously described with labeling efficiency 80–90% [16]. The pyrene-labeled PA_83_ protein was treated with trypsin and then purified as (PA_63_)_7_ heptamer with ion-exchange chromatography. 3 nM of the pyrene-labeled (PA_63_)_7_ was incubated with 40 nM of R218, R318, or the C/A mutants of R318 at pH 8.5 for 30 min. Conversion of prepore to pore in solution was triggered by addition of 1/10 volume of 1 M sodium acetate (pH 5.0). Pyrene fluorescence emission was recorded at 475 nm with excitation at 345 nm. Rates of Pyrene fluorescence were calculated in SigmaPlot by fitting the curves to the single exponential equation f = a*[1 - exp(- b*x)], in which b is the rate of fluorescence emission.

### Liposome preparation

Liposomes were prepared as previously described [26,27,29,30]. Briefly, 1,2-Dioleoyl-sn-glycero-3-phosphocholine (DOPC) (Avanti Polar Lipid) was mixed with Ni^2+^-chelating lipids, DOGs-NTA-Ni (1,2-dioleoyl-sn-glycero-3-{[N(5-amino-1-carboxypentyl) iminodiacetic acid]succinyl} nickel salt) in a molar ratio 100:8 in chloroform (Avanti Polar Lipids). The lipid was dried under N_2_ gas to form a lipid film, followed by vacuum for 3 h to remove residual solvent. The dried lipid film was rehydrated with the buffer containing 5 mM of the dye/quencher pair 8-aminonapthalene-1,3,6 trisulfonic acid (ANTS)/p-xylene-bis-pyridinium bromide (DPX) to form multilamellar vesicles and subjected to three freeze-thaw cycles and extrusion through a 200-nm pore size polycarbonate filter (Nucleopore) in a mini extruder (Avanti Polar Lipids). The protocol yielded large unilamellar vesicles with an average diameter of 150–200 nm.

### Time-lapse intensity measurement of NBD emission

PA_83_ proteins carrying the mutation G305C or N306C were labeled with N,N'-dimethyl-*N*-(iodoacetyl)-*N’*-(7-nitrobenz-2-oxa-1,3-diazol-4-yl) ethylenediamine (IANBD), as previously described with labeling efficiency of 80–90% [29,30]. The NBD-labeled PA_83_ proteins were treated with trypsin and then purified as (PA_63_)_7_ heptamers. NBD emission of PA pores in the liposomal membranes was measured as previously described [29,30]. Briefly, 3 nM of (PA_63_)_7_ was pre-incubated with 40 nM of R218-His_6_, R318-His_6_, or the C/A mutants of R318-His_6_ at pH 8.5 for 30 min, and then incubated with the Ni^2+^-liposomes for another 30 min. The protein-liposome mixtures were transferred to a cuvette with a stirring bar in the ISS K2 fluorometer. NBD was excited at 488 nm, and emission was recorded at 544 nm. Crossed polarizers on excitation, emission beams, and a 520-nm filter were used to reduce the background scatter. After addition of 1/10 volume of 1 M sodium acetate (pH 5.0) to the cuvette, the shift of NBD label from a polar (solution) to a nonpolar environment (liposomal membrane) was monitored.

### ANTS/DPX fluorescence dequenching

The ANTS fluorescence dequenching assay for PA pore formation was developed as previously described [27,31]. Briefly, 3 nM of (PA_63_)_7_ was pre-incubated with 40 nM of R218-His_6_, R318-His_6_, and the indicated C/A mutants in 20 mM Tris-HCl (pH 8.5), 150 mM NaCl, 1mM MgCl_2_, at room temperature for 30 min. Then, 100 μl of the liposomes doped with ANTS/DPX were incubated with the protein mixture for an additional 30 min. The protein/liposome mixture was diluted into 1.3 ml of 20 mM TrisHCl (pH 8.5), 150 mM NaCl, 1 mM MgCl_2_ with continuous stirring in the ISS-K2 fluorometer with excitation at 380 nm and emission at 520 nm. After the base line was stabilized, 300 μl of 1M sodium acetate (pH 5.0) was injected into the cuvette, and the fluorescence signal was monitored in real-time. Crossed polarizers on excitation and emission beams, and a 435-nm long path filter were used to reduce the background scatter. Rates of fluorescence dequenching were calculated in SigmaPlot by fitting the curves to the single exponential equation f = a*[1 - exp(- b*x)], in which b is the rate of fluorescence dequenching.

### Homology modeling of the Ig-like domain of ANTXR2

The sequence of the Ig domain (residues 219–318) was submitted to the I-TASSER server for automated homology modeling [32]. Five models were generated, and the first model with the highest C-score of -0.88 was chosen. The top template was PDB 2CXK. A similar model was obtained by using SWISS-MODEL [33].

### EM specimen preparation and data collection

3.5 μl of the purified protein complexes PA-R318, PA-TF-R318, PA-TF-R318(4C/A), and PA-R318(C255/279A) were blotted and negatively stained on ultrathin carbon films (Ted Pella #01824) using 2% sodium phosphotungstate (pH 7.4). The data were collected at 300 kV with a 100,000 x nominal magnification on a JEOL JEM-3200FS transmission electron microscope, which is equipped with a field emission gun and an in-column omega filter. The micrographs were recorded on a Gatan 4k x 4k UltraScan US4000 CCD camera with defocus values listed in Table 1. The final calibrated magnification is 117,188 that equal to a pixel size of 1.28 Å on the micrograph. 2 x 2 pixel binning was applied to obtain a final pixel size of 2.56 Å for image processing.

**Table 1:** EM Data collection and image processing of negatively stained samples.

Samples	Defocus (μM)	Micrographs (Used)	Particles (Used)	Resolution (Å)
PA-TF-R318	0.53–1.82	28	3784	17.4
PA-TF-R318 (4C/A)	0.89–1.74	45	3746	16.2
PA-R318	0.53–1.50	65	3387	13.8
PA-R318(C255/279A)	0.52–1.52	85	3852	13.1

#### Image processing, 3D reconstruction, and docking of atomic models

Image analysis and 3D reconstruction were carried out using EMAN imaging suites. The particles were automatically selected but visually checked using EMAN1 [34] and later using EMAN2.1 [35]. The data were linearized and normalized using EMAN2.1. Judged by image Fourier transfer, only the particles selected from the micrographs without visible drift and with minimal astigmatism were used in the reconstructions. The defocus of each micrograph was determined by CTFit. Phase flipping was applied during reconstruction to correct contrast transfer function (CTF). The reference-free initial model was generated using EMAN2.1. The maps were first refined briefly using EMAN2.1 and then refined by FREALIGN [36], which generated better maps. The maps were refined until their resolutions were not improved. The final 3D reconstruction for each dataset was computed from a number of particles listed in Table 1. The resolution of each map was determined with Fourier shell correlation (FSC) at a cutoff of 0.5. The EM maps were docked with the X-ray crystal structure of the VWA domain-bound PA heptamer (PDB 1TZN) using the “Fit in map” function in UCSF Chimera [37]. The I-TASSER homology modeled Ig domain was also docked into the EM map of PA-R318 heptameric complex using Chimera and linked to the VWA domain. The linkage loop region between VWA domain and Ig domain was further refined through iterative cycles of energy minimization in Chimera.

## Results

### The residues C255/C279 and C230/C315 form two disulfide bonds in the Ig domain of ANTXR2

**Fig 1:**
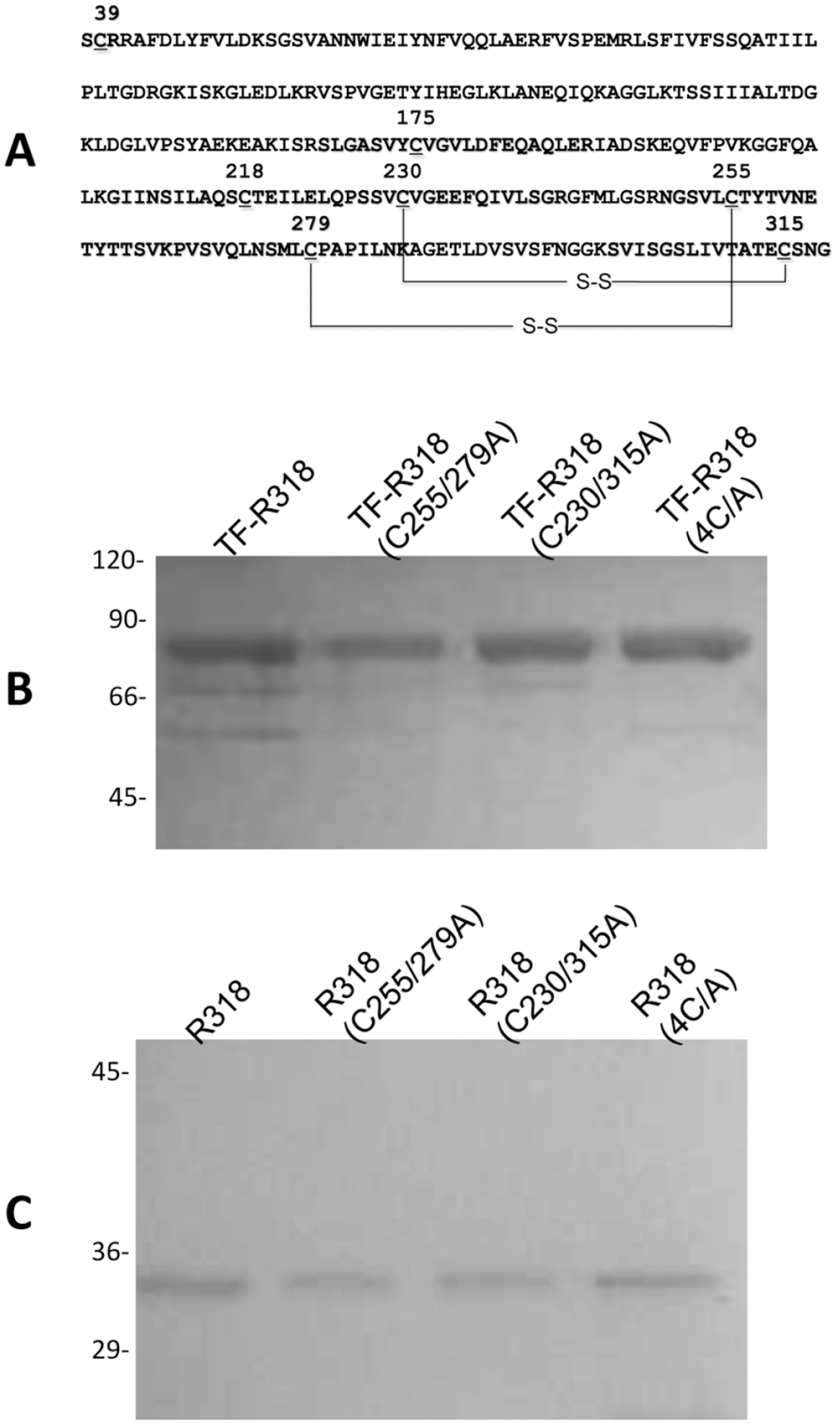
The residues C255/C279 and C230/C315 form two disulfide bonds in the Ig domain of ANTXR2. **A.** In the ANTXR2 ectodomain (residues 38–318), C255/C279 and C230/C315 form two disulfide bonds in the Ig domain (residues 219–318). **B.** The purified TF-R318 and the C/A mutants of TF-R318 as indicated were run in SDS-PAGE, stained by Coomassie blue. **C.** R318 and the R318 C/A mutants were purified after removal of TF tags and run in SDS-PAGE, followed by Coomassie blue staining.

In an earlier study using Ellman’s reagent, we have found that C175 is the only free Cys residue in the purified ANTXR2 ectodomain (here termed R318) [26]. Thus, the other six Cys residues presumably form three disulfide bonds. Since C39 and C218 form a disulfide bond in the crystal structure of the VWA domain (termed R218) [15], it is predicted that four Cys residues in the Ig domain form two disulfide bonds (Fig 1A). To determine the exact disulfide pairing in the Ig domain, we generated a series of mutations by replacing Cys with Ala two at a time in all possible combinations: C230A/C255A, C230A/C279A, C230A/C315A, C255A/C279A, C255A/C315A, C279A/C315A. As a control, we also replaced all of the Cys residues with Ala, which generated the 4C/A mutant. Following the previously described procedures [27], we expressed and purified the recombinant receptor ectodomains with respective C/A mutations in *E*. *coli*. While all of the proteins were expressed at a similar level in *E*. *coli*, only the proteins with mutations C230A/C315A, C255A/C279A and 4C/A were purified as soluble monomeric proteins (Fig 1B and 1C). The rest of the mutants failed to be purified, because they formed aggregates in the cells, which was primarily due to misfolding and/or disulfide crosslinking (data not shown). Moreover, liquid chromatography-tandem mass spectrometry (LC-MS/MS) analysis of the purified R318 recovered 55% of the sequence, in which two trypsin/chymotrypsin-digested polypeptides were linked with a disulfide bond formed by C255 and C279 (S1 Fig). Together, this result suggests that C230/C315 and C255/C279 form two stable disulfide bonds in the Ig domain (Fig 1A). The disulfide linkage is consistent with the results in a recent report [38], in which C230/C315 and C255/C279 were predicted to form two disulfide bonds by homologous modeling and by expression of the receptor genes containing C/A mutations in mammalian cells.

### The Mutations of C255A/C279A, but not C230A/C315A, inhibited the PA pore-induced dequenching of ANTS fluorescence

**Fig 2:**
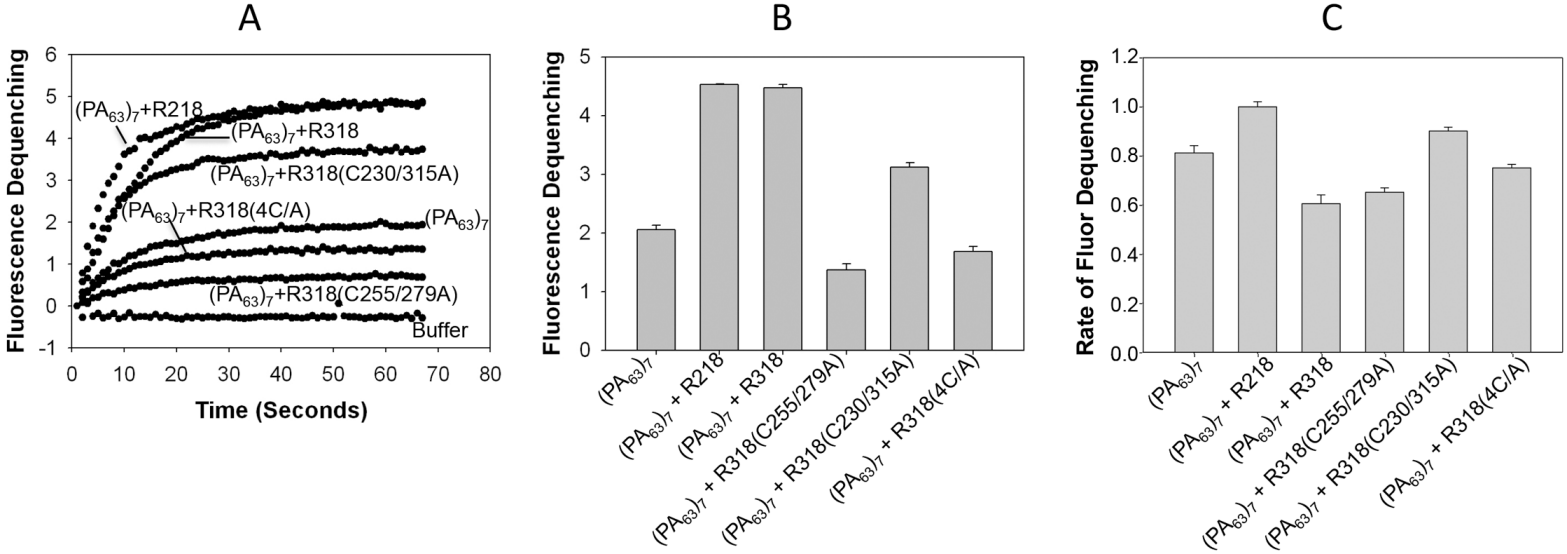
Mutations of C255A/C279A, but not C230A/C315A, inhibited the PA-induced ANTS fluorescence dequenching in the liposomes. 3 nM of heptameric prepore (PA_63_)_7_ was mixed with 40 nM of the purified receptor domains as indicated, and then incubated with the liposomes doped with ANTS/DPX. Pore formation was triggered by acidification, and ANTS dequenching was measured at 435 nm with excitation at 380 nm. The representative curves of ANTS fluorescence were shown in **A**. Relative ANTS fluorescence intensity at 60 seconds of post-acidification was quantified from three independent repeats and shown in **B**. The relative rates of fluorescence dequenching were calculated in SigmaPlot as described in Materials and Methods and shown in **C**.

To measure PA pore function, we recently adopted a sensitive, convenient ANTS/DPX fluorescence dequenching assay as a replacement of the K^+^ release assay that was previously established in our laboratory [27,31]. ANTS/DPX, the anion/cation fluorophore/quencher pair, is widely used for membrane leakage. ANTS fluorescence is quenched by DPX inside the liposomes, and it is dequenched upon release into the medium. We have shown that ANTX/DPX fluorescence-dequenching assay faithfully replicated the results obtained from the K^+^ release assay [27,31]. Here we used this assay to determine effects of disulfide deletion on functioning of PA pore (Fig 2). In the absence of receptor, PA alone caused a low level of ANTS fluorescence dequenching, while the PA complexed with either R218 or R318 induced significantly higher fluorescence (Fig 2A and 2B). The result was consistent with the previous findings that PA pore formation was greatly enhanced when PA was pre-bound to the liposomal membranes through binding of the His-tags on the receptor domains to the Ni^2+^-chelating lipids on the liposomal membranes [26,27,29]. However, when PA was bound to disulfide deletion mutants either R318(C255/279A) or R318(4C/A), the dequenching of ANTS fluorescence was drastically inhibited, to a level that is even lower than that of PA alone (Fig 2A and 2B). Again, this result is consistent with our previous findings, where reduction of R318 by TCEP or DTT inhibited the PA-induced K^+^ release from liposomes [26]. Compared to C255A/C279A and 4C/A, mutations of C230A/C315A only have a partial effect on dequenching of ANTS fluorescence (Fig 2A and 2B). Together, these data suggest that the disulfide bond C255-C279 is required for functioning of PA pore. Interestingly, while deletion of the disulfide bond C255-C279 significantly reduced the level (amplitude) of fluorescence dequenching, it did not affect the kinetics (the time to reach the plateau) as shown in Fig 2C, where R318(C255/279A) has a similar rate with R318(WT). Deletion of the disulfide bond C255-C279 also did not affect the binding affinity between receptor and PA at neutral pH (S2 Fig). Taken together, these results suggest that deletion of the disulfide bond C255-C279 reduces the efficiency (the number) of the receptor-bound PA prepores to form a functional pore on the membrane, but not the kinetics of pore formation.

### Deletion of the disulfide bonds did not affect PA prepore-to-pore conversion

**Fig 3:**
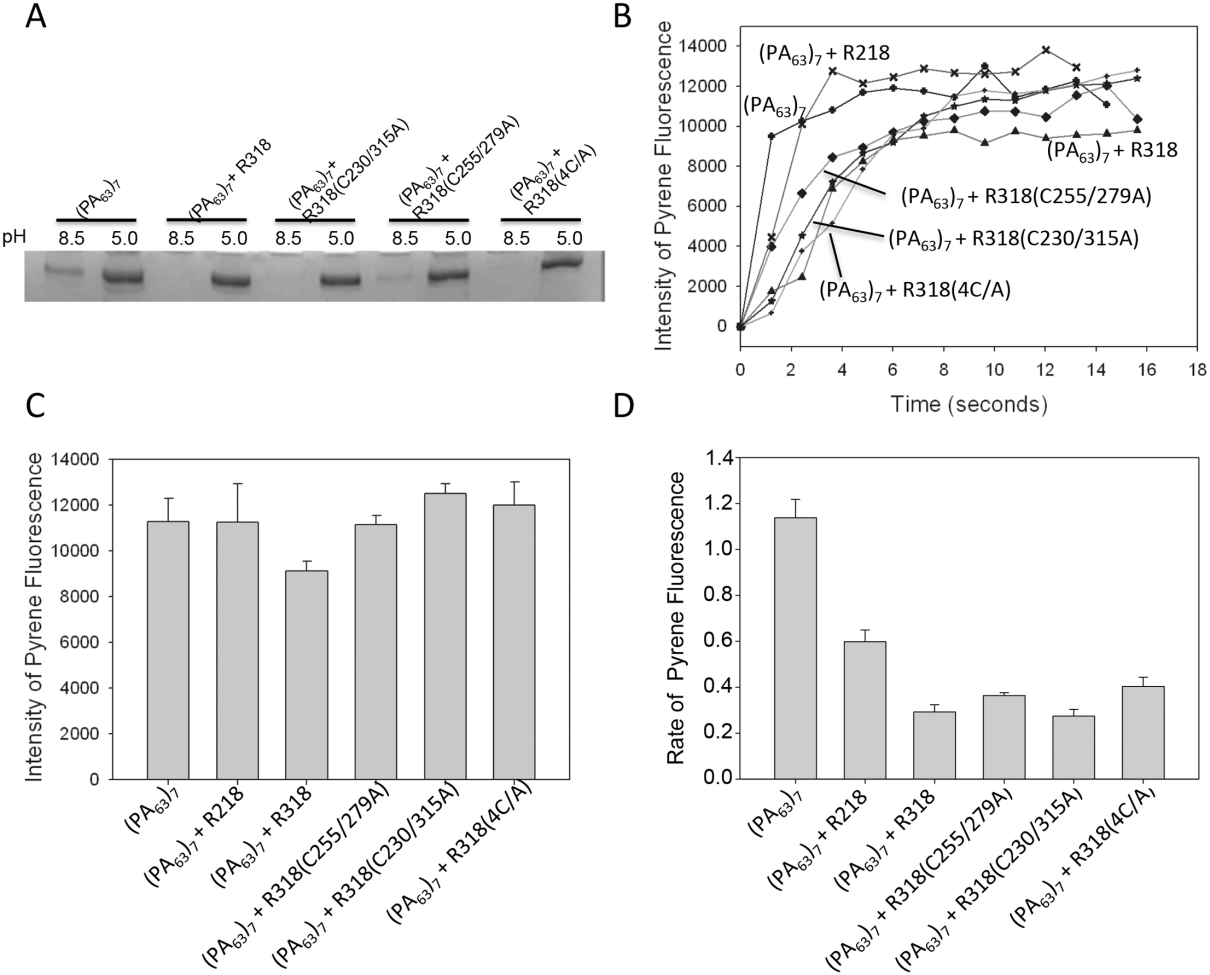
Disulfide deletion did not affect PA prepore-to-pore conversion. **A.** 5 μg of (PA_63_)_7_ was incubated with a 3-fold molar concentration of the purified receptor domains as indicated in pH 8.5 buffer for 30 min in the presence of 1 mM MgCl_2_. The pH of the buffers either remained constant or dropped to pH 5.0. Subsequently, the formation of SDS-resistant oligomers was examined in SDS-PAGE, followed by Coomassie blue stain. **B**. 3 nM of the pyrene-labeled PA(N306C) heptamer was mixed with 40 nM of R218, R318 and the R318 C/A mutants as indicated in pH 8.5 buffer. Conversion of prepore to pore was triggered by acidification (pH 5.0) and measured by pyrene fluorescence at 475 nm with excitation at 345 nm. The representative curves of pyrene fluorescence were shown. **C**. The relative intensity of pyrene fluorescence at 15 seconds of post-acidification was calculated from three independent measurements. **D.** The relative rates of pyrene fluorescence were calculated in SigmaPlot as described in Materials and Methods.

Next, we sought to test effects of the disulfide deletion on PA prepore-to-pore conversion. Formation of SDS-resistant oligomers in SDS-PAGE is a well-documented marker for PA prepore-to-pore conversion [4,18,39]. Previously, we have found that reduction of the receptor by TCEP did not affect PA prepore-to-pore conversion either in solution or on the cell surface membranes [26]. Consistent with this result, here we found that deletion of either C255-C279 or C230-C315, or both, did not affect formation of the SDS-resistant oligomer at acidic pH (Fig 3A). To further confirm effects of the disulfide deletion on PA prepore-to-pore conversion, we used pyrene fluorescence to monitor the PA conformational transition in real time. Pyrene is a spatially sensitive extrinsic fluorescent probe, which forms excited-state complexes (excimers) upon close encounter with other pyrene molecules. In a previous study, pyrene maleimide was attached to the residue N306C located on the PA 2β2–2β3 loop. The pyrene labels are too far apart in the prepore structure to interact, but their proximity within the β-barrel of the pore leads to excimer formation and fluorescence [16]. Here, we monitored the prepore-to-pore conversion using the pyrene-labeled PA(N306C) that was bound to the receptor domains as indicated (Fig 3B). Similar to the results of SDS-resistant oligomer formation (Fig 3A), PA that is either free or bound with various receptor domains exhibited similar levels of pyrene fluorescence (Fig 3B and 3C), suggesting that deletion of the disulfide bonds did not inhibit prepore-to-pore conversion. Next, we calculated the rates of pyrene fluorescence (Fig 3D). Relative to PA alone, the R218-bound PA has a slower rate of prepore-to-pore conversion, which is consistent with the role of the VWA domain functioning as a molecular switch that only allows PA prepore to convert into pore below the pH threshold. Interestingly, the R318-bound PA has a slower rate than the R218-bound PA, suggesting that the additional Ig domain may also play a role in prepore-to-pore conversion. Compared to R318(WT), deletion of any of the disulfide bonds did not affect the kinetics of PA prepore-to-pore conversion, which is consistent to the result in Fig 2C.

### Deletion of the disulfide bond C255-C279 blocked membrane insertion of PA pore

**Fig 4:**
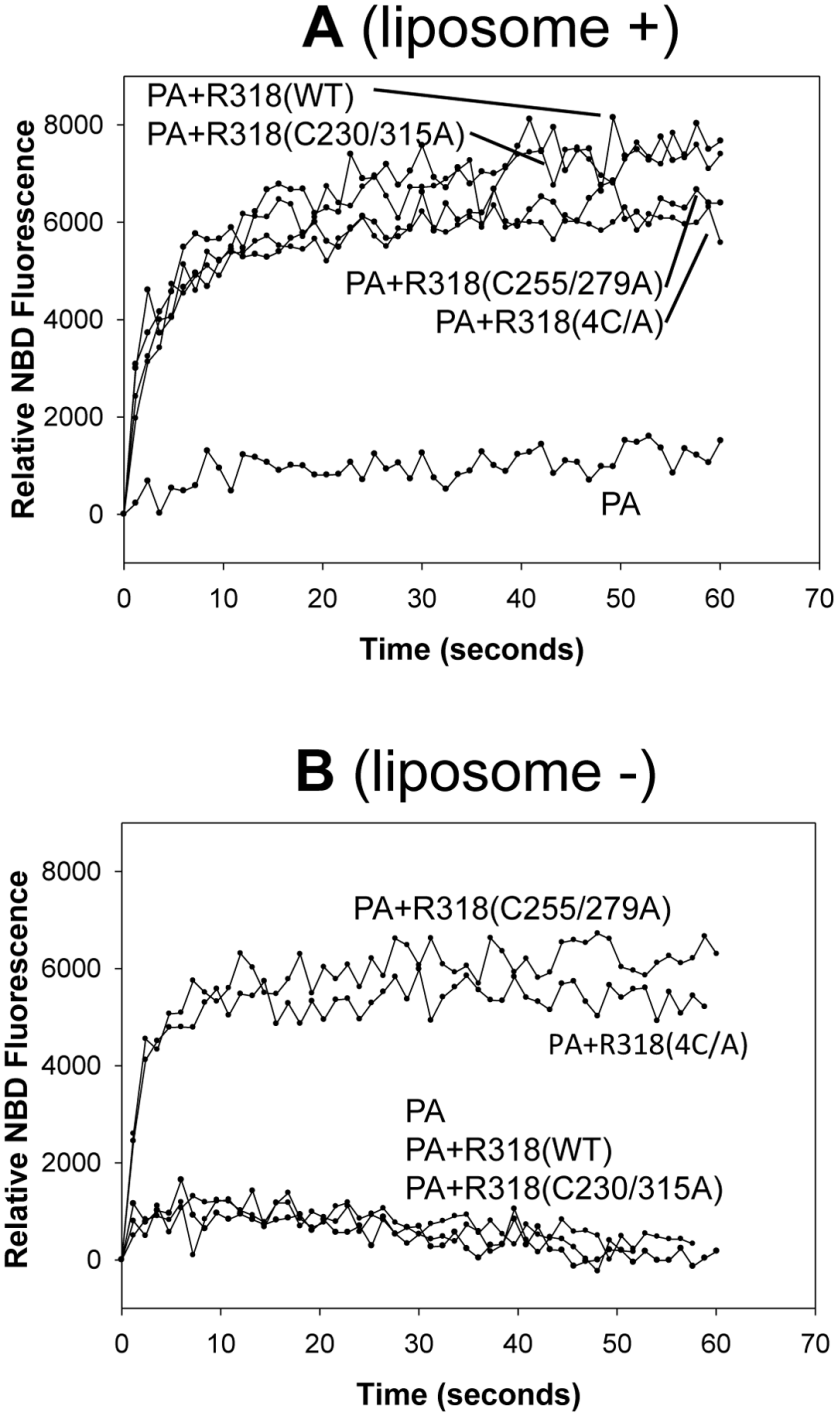
Deletion of the disulfide bond C255-C279 inhibited membrane insertion of the PA pore. **A.** 3 nM of NBD-labeled PA(G305C) heptamer was first bound to the receptor domains as indicated in pH 8.5 buffer. Then the PA-receptor complexes were incubated with the liposomes containing Ni-chelating lipids on the membranes. Membrane insertion was triggered by acidification (pH 5.0). The NDB fluorescence was measured at 544 nm with excitation at 488 nm. **B.** As controls, the NBD-labeled PA-receptor complexes were acidified without the presence of liposomes. The representative curves from three independent measurements were shown.

Since disulfide deletion did not affect PA prepore-to-pore conversion, we tested if it affects PA pore membrane insertion. NBD is an environment-sensitive dye. With excitation at 488 nm, the emission intensity of NBD fluorescence at 544 nm increases substantially upon a shift from a polar (aqueous) to a non-polar (lipid) environment. Here we used NBD fluorescence to test the effects of disulfide deletion on PA pore insertion on the liposomal membranes. As previously described [29,30], NBD was attached to G305C, a lipid-facing residue of the PA pore. As expected, PA alone showed little NBD fluorescence, while the R318-bound PA showed a significantly higher NBD fluorescence. Surprisingly, however, the mutants with deletion of the disulfide bonds either C255-C279 or C230-C315, or both, produced comparable levels of NBD fluorescence (Fig 4A). Since the increase in NBD emission could result from either insertion into lipid membranes or insertion into hydrophobic pockets of proteins, we tested if the observed NBD emission is dependent on the presence of liposomes. As expected, in the absence of liposomes, the R318(WT)- and R318(C230/315A)-bound PA exhibited little NBD emission. Interestingly, however, the R318(C255/279A)- and R318(4C/A)-bound PA still showed strong NBD emission, suggesting that the observed NBD emission resulted from insertion into proteinaceous hydrophobic pockets (Fig 4B). Thus, deletion of the disulfide bond C255-C279 inhibited PA insertion into the membranes by trapping the PA membrane-inserting loops in proteinaceous hydrophobic pockets.

### Reduction or deletion of the disulfide bond C255-C279 resulted in a significant conformational change on the receptor ectodomain

**Fig 5:**
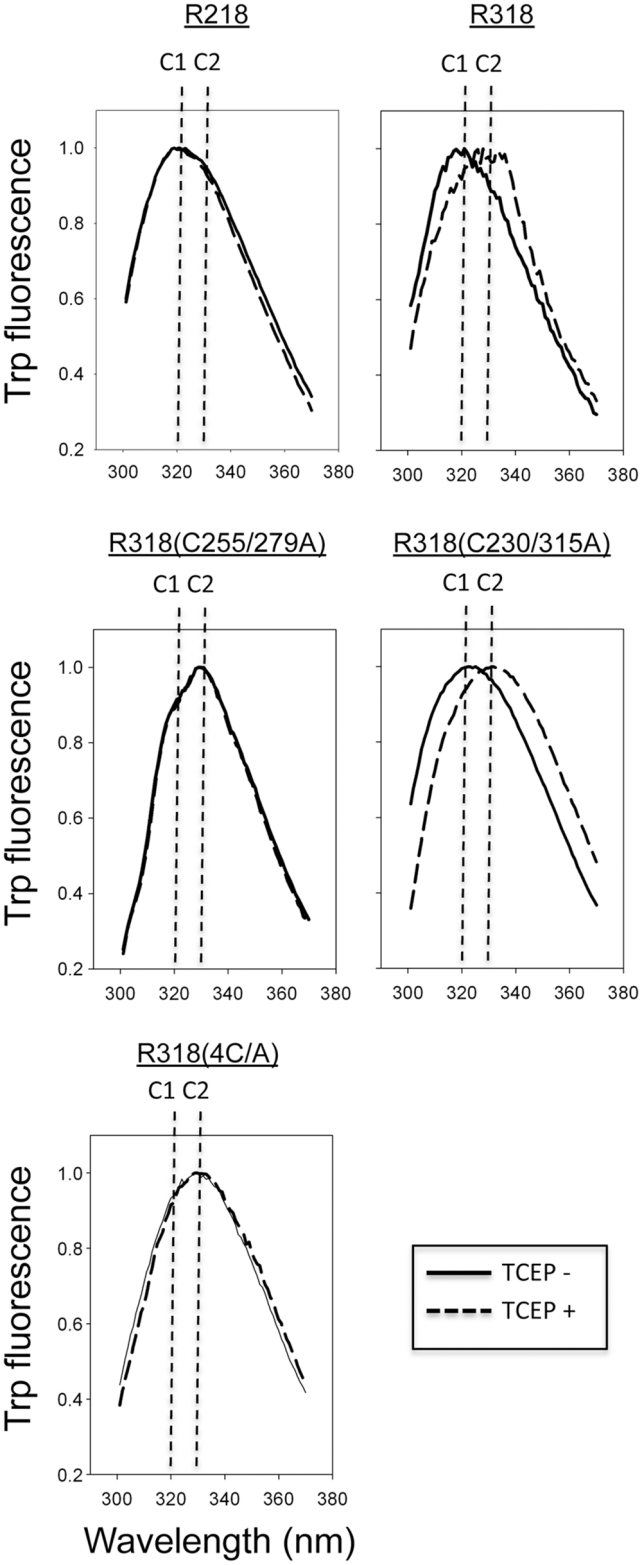
Mutations of C255A/C279A, but not C230A/C315A, resulted in a significant conformational change on the receptor ectodomain. 5 μM of the purified receptor domains were incubated in the 20 mM Tris-HCl (pH 7.3), 100 mM NaCl, in the presence or absence of 5 mM TCEP. The intrinsic Trp59 fluorescence spectrum was measured with excitation at 290 nm and emission at 300–370 nm. Note: two distinct spectrum peaks (320 nm and 330 nm) were detected and represented as two distinct conformations, denoted as C1 and C2.

Trp59, the only Trp residue in the ectodomain, is located at the top of the VWA domain, adjacent to the PA-binding interface. Here, we tested the intrinsic fluorescence of Trp59 to monitor the conformational changes induced by disulfide disruption. The spectra of Trp59 fluorescence on R218, R318, R318(C230/315A), R318(C255/279A), and R318(4C/A) were measured in the presence or absence of TCEP (Fig 5). The Trp59 spectrum of R218 is not responsive to TCEP treatment, which is consistent with the previous finding that C39-C218 is dispensable for maintaining the VWA structure [15]. R318 underwent a significant conformational change upon TCEP treatment, evidenced by a shift of the spectrum peak from 320 nm to 330 nm. R318(C230/315A) displayed spectra that were similar to R318, suggesting that deletion of the disulfide bond C230-C315 did not affect the receptor conformation. However, R318(C255/279A) without TCEP treatment has a spectrum peak at 330 nm, and it made no additional conformational change upon TCEP treatment, indicating that deletion or reduction of the disulfide bond C255-C279 was responsible for the receptor conformational change. As predicted, R318(4C/A) had a spectrum peak at 330 nm in the presence or absence of TCEP. The result clearly demonstrated that the disulfide bond C255-C279, in either forming or breaking, is responsible for two distinct conformations of the receptor ectodomain, Conformation 1 (C1) with the spectrum peak of Trp59 at 320 nm or Conformation 2 (C2) at 330 nm.

### Single-particle EM reconstruction located the Ig domain in the heptameric complex of PA-R318 and detected the disulfide deletion-induced conformational change on the VWA domain

**Fig 6:**
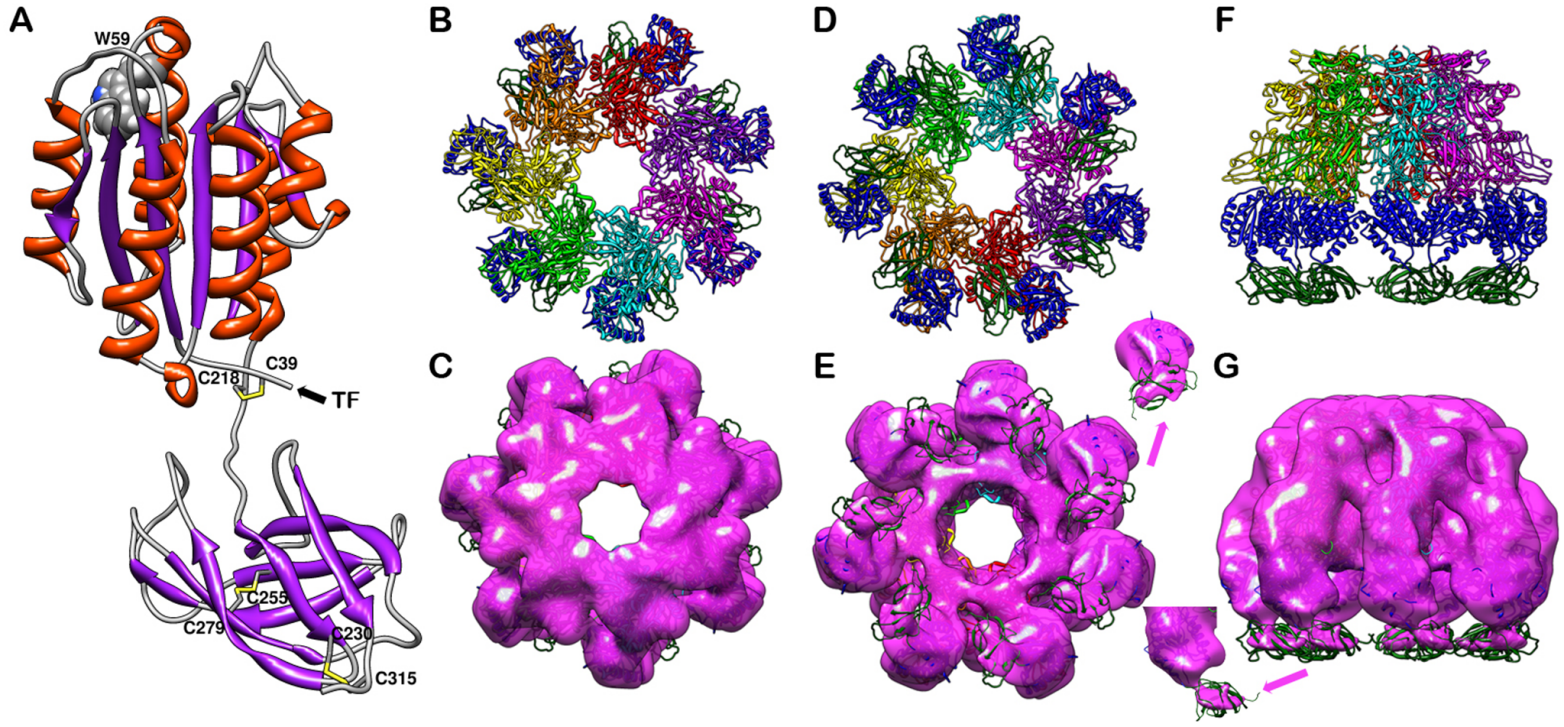
Homology modeling of the Ig domain and docking of the atomic structure into the reconstructed EM maps. **A**. The structure of the Ig domain is generated by homologous modeling and grafted to the crystal structure of the VWA domain through energy minimization. The secondary structure is colored as α-helices in orange, β-sheets in purple and loops in grey. The disulfide bonds C39-C218, C255-C279 and C230-C315 are shown as stick models, colored in yellow. Note: Trp59 is shown in a sphere model and labeled. **B**, **D**, and **F** are ribbon diagrams of PA-R318 heptameric complex viewed from top, bottom and side, respectively. **C**, **E**, and **G** are surface rendered density maps from reconstruction of negatively stained PA-R318 heptameric complex, docked with the modeled structure and viewed from top, bottom and side, respectively. Segmented map density of one subunit of the receptor ectodomain is shown on up-right in **E** or lower-left in **G**, respectively. The map was rendered at the level of one standard deviation above the average density value of the map. Within the PA heptamer, each of the seven monomers are colored in red, orange, yellow, green, cyan, magenta, and purple, respectively. The VWA domains and the Ig domains are colored in blue and green, respectively. The reconstructed EM maps are rendered in magenta.

While *in silico* analysis has indicated that the residues 219–318 form an Ig fold [26,38], its structure and position in the PA-receptor heptameric complex are still unknown. Using single-particle 3D reconstruction of negatively stained samples, we determined a low-resolution map (~14Å) of PA-R318 heptameric complex (Fig 6C, 6E and 6G). In parallel, we generated a structural model of ANTXR2 ectodomain by homology modeling (Fig 6A). The modeled Ig domain in combination with the crystal structure of PA-VWA heptameric complex was docked in the reconstructed EM map (Fig 6B–6G). Apparently, the modeled Ig domain fit into the additional density underneath the complex. We also noticed that the density of the Ig domain was significantly weaker than that expected by molecular weight and only appeared when the contour level of the map dropped to one standard deviation above the average density level, whereas the other part of the complex could be rendered at three and half standard deviation above the average density level and still fit the atomic model very well. This suggests that the Ig domain is flexible in solution compared to PA and the VWA domain. This is consistent with the structural model, in which the hinge region between VWA and Ig allows certain flexibility between the two domains.

**Fig 7:**
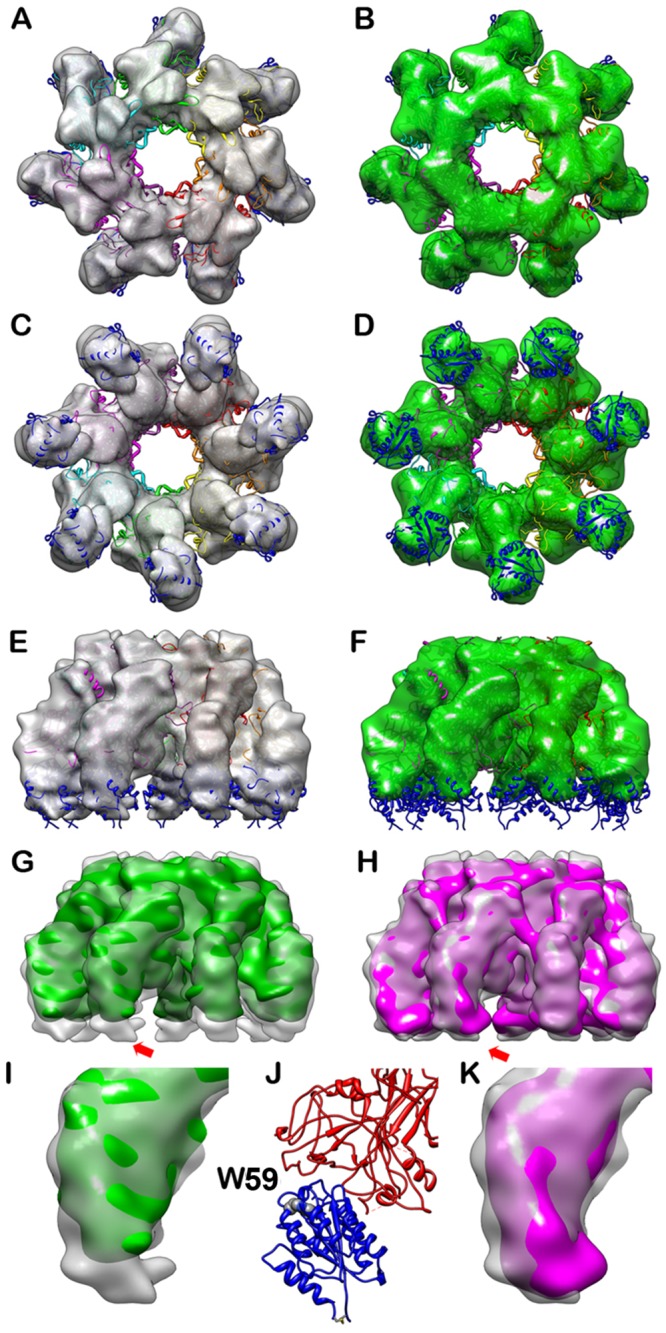
3D reconstruction of negatively stained PA-TF-R318 and PA-TF-R318(4C/A) detected the disulfide deletion-induced conformational changes on the VWA domain. **A**, **C**, and **E** are surface rendered density maps of PA-TF-R318 heptameric complex viewed from top, bottom and side. **B**, **D**, and **F** are surface rendered density maps of PA-TF-R318(4C/A) viewed from top, bottom and side. The crystal structure of the PA-VWA heptameric complex was docked in the reconstructed maps. **G** is the side view of the superposed density maps from PA-TF-R318 (transparent grey) and PA-TF-R318(4C/A) (solid green). **H** is the side view of the superposed density map from PA-TF-R318 (transparent grey) and PA-R318 (solid magenta) showing high similarity of both maps. **I** and **K** are the zoom-in monomeric view for the area at the lower part of the complexes as show in **G** and **H** respectively. **J** is the ribbon diagram of the fitted PA and VWA structure at the same orientation and magnification as in **I** and **K**. All of the maps were rendered at the level of three and half times standard deviation above the average density value of the maps. The PA is colored in red and the VWA is colored in blue. Trp59 of VWA is rendered as space-filling model and labeled.

To observe the conformational changes induced by disulfide disruption, we first reconstructed the EM maps for the heptameric complexes of PA-TF-R318 and PA-TF-R318(4C/A) (Fig 7A–7F). Trigger factor (TF) is an *E*. *coli* chaperon that has been shown to improve the solubility and stability of R318 proteins, especially for the R318 Cys/Ala mutants [27]. Moreover, TF tag at the N-terminus of R318 did not affect the function of the receptor in mediating PA pore formation (data not shown). More importantly, TF tags appeared to increase the visibility and provide more orientations of the protein particles on the EM grids after negative staining. Surprisingly, however, in the reconstructed EM map of PA-TF-R318 we could not locate the density of Ig domains and TF tags (Fig 7A, 7C and 7E). We noticed that in the pCOLD-TF vector there is an extended polypeptide linker (26 residues in total) between TF and the inserted gene. This long linker may cause significant flexibility between TF and receptor, which made density mapping of the structures beneath the VWA domains difficult to interpret, especially when the maps were rendered at a low contour level. Therefore, we rendered the map of PA-TF-R318 at a density level of three and half standard deviation above the average value to remove the disordered density beneath the VWA domains. The resultant map of PA-TF-R318 fits the crystal structure of the PA-VWA heptamer very well (Fig 7A, 7C and 7E). Using the same parameters, we reconstructed the EM map of PA-TF-R318(4C/A) (Fig 7B, 7D and 7E). Compared to the map of PA-TF-R318, PA-TF-R318(4C/A) apparently has less density in the VWA domain (Fig 7G, 7I and 7J), suggesting that the VWA domain on the 4C/A mutant was either partially unfolded and/or became more flexible. This observation is consistent with the data from Trp59 fluorescence experiment. To rule out the possibility that TF tags may interfere with the density mapping, we compared the reconstructed maps of PA-R318 and PA-TF-R318 by superimposing the two maps rendered at a three and half standard deviation above average density level (Fig 7H and 7K). The figures show that there is no significant difference between the two maps, suggesting that the presence of the flexible TF tags did not affect the density mapping of PA and VWA.

**Fig 8:**
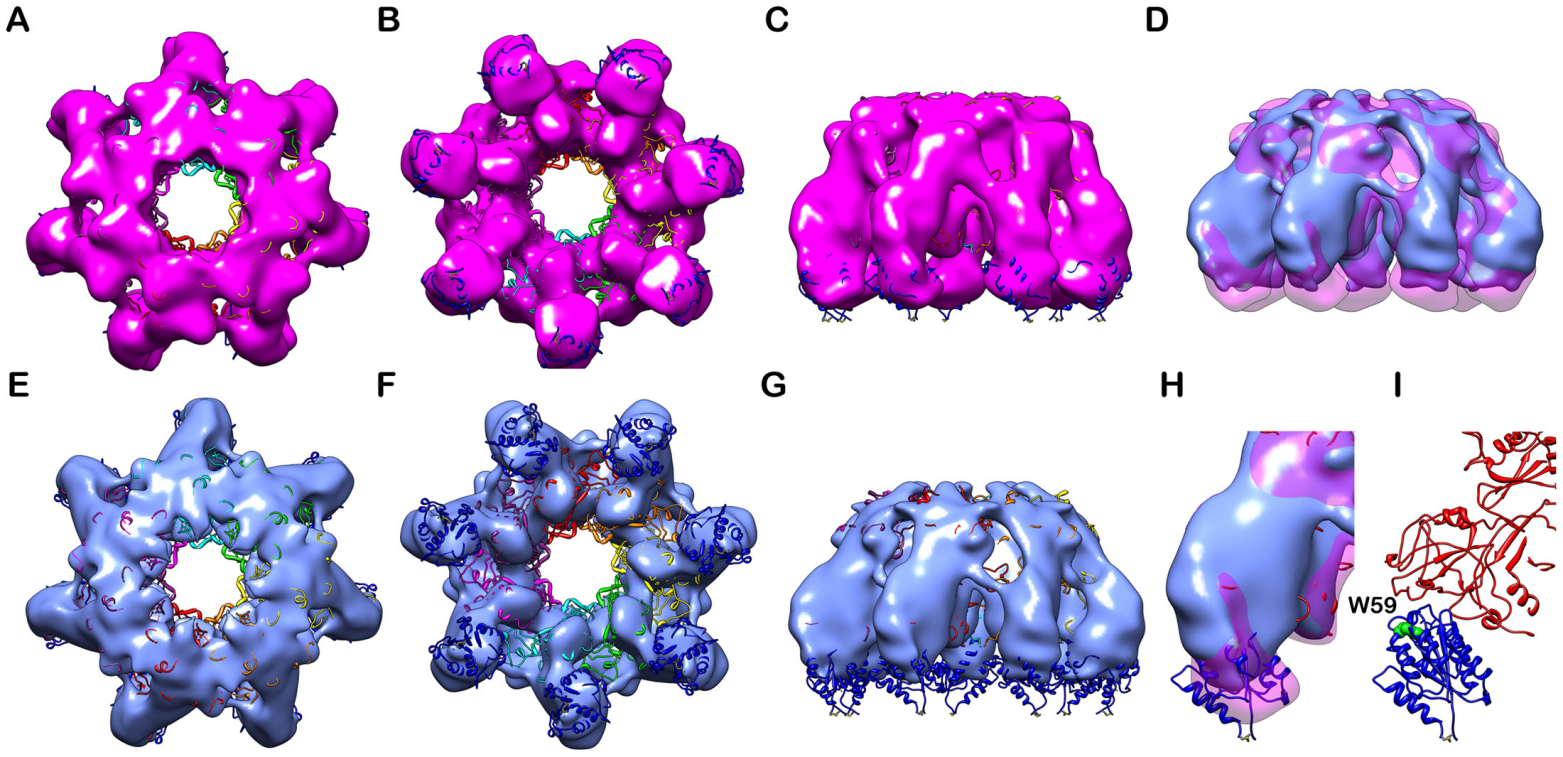
Compared to PA-R318, PA-R318(C255/279A) exhibited a significant conformational change on the VWA domain. **A**, **B**, and **C** are surface rendered density maps of PA-R318 heptameric complex viewed from top, bottom and side in magenta. **E**, **F**, and **G** are surface rendered density maps of PA-R318(C255/279A) viewed from top, bottom and side in cyan. The crystal structure of the PA-VWA heptameric complex was docked in the reconstructed maps. **D** is the side view of the superimposed density maps from PA-R318 (transparent magenta) and PA-R318(C255/279A) (solid cyan). **H** is the side view of the superimposed density map from PA-R318 (transparent magenta) and PA-R318(C255/279A) (solid cyan) that shows the missing densities in the VWA domain. **I** is the ribbon diagram of the crystal structure of the PA-VWA heptamer at the same orientation and magnification as in **H**. All of the maps were rendered at the level of three and half times standard deviation above the average density value of the maps. The PA is colored in red and the VWA is colored in blue. Trp59 of VWA is rendered as spherical model and labeled.

To further confirm the observed conformation change in R318(C255/279A) without TF tag, we later obtained the negatively-stained EM map of the heptameric complex PA-R318(C255/279A) (without TF tag) (Fig 8). As expected and similar to PA-TF-R318(4C/A), PA-R318(C255/279A) has less density in part of the VWA domain when compared with PA-R318. In summary, all the biochemistry and structural data convincingly demonstrated that deletion of the disulfide bond C255-C279 induced significant conformational changes in both the Ig domain and VWA domain.

## Discussion

Our previous study has shown that four Cys residues form two disulfide bonds in the Ig domain, and reduction of these disulfide bonds inhibited the PA-mediated release of K^+^ ions from liposomes and CHO cells, and also inhibited the PA-mediated translocation of the model substrate LF_N_ across the cell membranes [26]. In the present study, we unambiguously identified that the disulfide bond C255-C279, but not C230-C315, is required for the receptor-bound PA pore to function, and furthermore we provided a mechanistic insight that deletion of the disulfide bond C255-C279 induces conformational changes on both Ig and VWA domains, which inhibits PA membrane insertion by sequestering PA membrane-inserting loops in proteinaceous hydrophobic pockets.

Current data clearly show that reduction or deletion of the disulfide bonds in the Ig domain do not disrupt apparent binding between R318 and PA. In our previous report, the reduced R318 remained bound to PA in a native gel electrophoresis. Moreover, in a reducing condition PA was still bound to the plasma membranes of the CHO cells that overexpress ANTXR2 [26]. In the present study, we obtained structural evidence that R318(C255/279A) or R318(4C/A) forms a stable complex with PA prepore (Figs 7 and 8). Finally, in SPR analysis we found that deletion of the disulfide bond C255-C279 did not reduce the binding affinity to PA at neutral pH (S2 Fig). Due to technical difficulty, we were not able to measure the affinity between receptor and PA during prepore-to-pore transition at acidic pH.

One can imagine that the observed inhibition of PA function could have resulted from the defects in one or more steps of the following PA actions: PA prepore-to-pore conformational conversion, PA pore membrane insertion, and PA pore-mediated substrate translocation. In our earlier report, reduction of the receptor disulfide bonds did not affect formation of SDS-resistant PA_63_ oligomers both in aqueous solutions and on the cell membranes [26]. Here, we showed that deletion of the disulfide bonds through mutagenesis, either C255-C279 or C230-C315, did not affect formation of PA_63_ SDS-resistant oligomers and pyrene fluorescence (Fig 3). Thus, we have clearly demonstrated that disulfide reduction or deletion did not inhibit prepore-to-pore conversion.

It is an interesting observation that the PA-R318(C255/279A) and PA-R318(4C/A) complexes exhibited liposome-independent NBD emissions, indicating that the NBD emission resulted from insertion of the NBD-labeled amino acid residues (G305C) into a proteinaceous hydrophobic pocket, instead of lipid membranes (Fig 4). Consistent to this finding, in our previous and present studies we repeatedly observed that upon disulfide reduction or deletion, the receptor-bound PA induced release of K^+^ or fluorescence dequenching from the liposomes at levels that were even lower than PA alone [26] (Fig 2). Taken together, these observations strongly suggest that reduction or deletion of the disulfide bond C255-C279 induces conformational changes on the receptor, which sequester the membrane-inserting loops of PA into hydrophobic pockets to prevent membrane insertion. This also explains the observation that deletion of the disulfide bond C255-C279 did not affect the kinetics of ANTS fluorescence dequenching (pore formation) and pyrene fluorescence (prepore-to-pore conversion) (Fig 2C and Fig 3D). We noticed that there were differences in kinetics of NBD emission for R318(C255/279A) and R318(4C/A) in the presence and absence of liposomes (S3 Fig). Multiple factors could contribute to the observed difference, such as solution hydrophobicity and/or light scattering due to the presence or absence of liposomes. Recent studies have supported a model of PA pore formation and membrane insertion, in which the 2β2–2β3 loop (amino acid residues 302–322) in the PA domain 2 moves to the base of the structure during the acidification-induced conformational rearrangement to form a 14-strand transmembrane β-barrel [28,40–42]. Thus, it is reasonable to believe that the PA membrane-inserting loops were intercepted and sequestered in the hydrophobic pockets formed by the partially unfolded Ig domain, or VWA domain, or both. Consistently, this mechanism of inhibition is supported by the evidence from Trp fluorescence (Fig 5) and single-particle EM analysis (Figs 6, 7 and 8), in which significant conformational changes in both the Ig domain and VWA domain were observed upon deletion or reduction of the disulfide bond of C255-C279. Therefore, this study has elucidated a novel molecular mechanism for inhibition of anthrax toxin action through disrupting a specific receptor disulfide bond. To inhibit anthrax toxin action, one can design and/or screen chemical compounds that directly target to and disrupt C255-C279, or through indirect mechanisms, such as modulation of cellular redox factors/enzymes, to affect the redox states of the receptor disulfide bonds.

While the crystal structures of the VWA domain and the heptameric PA-VWA complex have been solved [15–17], a high-resolution structure of the whole receptor ectodomain is currently lacking. It is largely due to technical difficulty in purification and crystallization of the whole ectodomain, especially because of the flexibility of the Ig domain that appears to affect stability and homogeneity of the samples. Single-particle EM analysis is a powerful tool in solving structures of large protein complexes. Our Cryo-EM attempts failed to achieve high-resolution reconstruction due to preferred orientations of the complexes within thin ice. Fortunately, the negatively stained samples on carbon supporting film did not show preferred orientations and allowed us reconstruct the low-resolution EM maps. During reconstruction of the negatively stained EM maps, we clearly noticed the flexibility of Ig domains, which suggests that, even if the problem of preferred orientations in Cryo-EM was solved, it was still not likely to achieve high resolution in these flexible regions. In combination with the biochemical analysis, however, the low-resolution reconstructions of negatively stained samples have provided adequate unambiguous structural information for the purpose of this study.

The EM map of PA-R318 is the first reported PA-ANTXR2 complex structure containing the under-studied Ig domain (Fig 6C, 6E and 6G). Thus, the EM map provides important information about the location and orientation of the Ig domain relative to VWA and PA within the complex. Linked with a flexible hinge region, the Ig domain is positioned underneath the VWA domain, with the disulfide bond C255-C279 located in the center of the Ig fold (Fig 6A). One can imagine that deletion or reduction of this disulfide bond will unfold the Ig domain. While C255-C279 appears to be far apart from Trp59 of the VWA domain, both Trp fluorescence and EM maps have suggested that deletion of the disulfide bond has a significant effect on the VWA domain (Figs 5, 7 and 8). This allosteric effect may be achieved through the unfolding of the Ig domain and/or the potential “breathing motion” between Ig and VWA caused by the flexible hinge. Moreover, within the PA-receptor complex, the Ig domains point towards to the center of the prepore lumen (Fig 6C, 6E and 6G). This close proximity could also potentially allow the Ig domains interfere with membrane insertion of the PA pore upon unfolding.

The results in the present study have suggested that R318(C255/279A) may remain bound to PA even after pore formation. However, it is not clear if R318(WT) remains bound to PA after pore formation. Current data have been controversial regarding PA-receptor binding after pore formation. An earlier study using a PA antiserum showed that both ANTXR1 and ANTXR2 were co-precipitated with the PA prepore, but not with the PA pore, suggesting receptors dissociate from PA upon pore formation [18]. Later, evidence obtained from the experiments using 1-dimensional NMR and 2-fluorohistidine labeled VWA domain supported the notion of receptor dissociation upon PA structural transition of prepore-to-pore [21]. Most recently, the kinetics of PA prepore-to-pore transition was measured with the immobilized complexes of PA prepore with LF_N_ using biolayer interferometry and surface plasmon resonance. When the soluble VWA domain was bound to the PA-LF_N_ complex, a decline in the magnitude of the signal following acidification was likely due to VWA dissociation from the pore. Moreover, VWA binding to the pH-transitioned PA pore was significantly weakened compared to the binding to PA prepore [22]. All of the above studies support the model of receptor dissociation upon PA pore formation. On the contrary, there is evidence supporting that receptors remain bound to PA pore after acidification. Co-immunoprecipitation using antibodies against the epitope tags on the cytosolic domain of ANTXR1 and ANTXR2 were able to co-precipitate both receptors with both PA prepore and pore [43,44]. A recombinant fragment of PA (PA domain 4) remained bound to the VWA domain at pH 5 [23]. A recent study using the transfer cross-saturation NMR approach demonstrated that the VWA domain contact with PA domain 2 is weakened prior to pore conversion, but the VWA domain remained bound to PA domain 4 following pore conversion, suggesting that the receptor plays a pore-stabilizing role [24]. In fact, the indication that the receptor plays an active role in influencing pore function were reported in an earlier patch clamp study of ion conductance by the PA pore in whole cells versus artificial membranes [45]. That study indicated that compared to the PA pore on the artificial membranes, the receptor-bound PA pore on the cell membranes has altered voltage-dependent inactivation properties and sensitivity to small molecular inhibitor TBA. In summary, more studies are required to probe the highly dynamic interaction between PA and ANTXR2 during and after prepore-to-pore conversion. Here, our studies using the whole ectodomain of the receptor with or without the disulfide bond C255-C279 have provided novel insights into this interaction.

Interaction between pathogens and their cellular receptors is a common scheme of host-pathogen interaction during viral and bacterial pathogenesis. There is increasing evidence that disulfide bonds are involved in pathogen-receptor interaction (e.g. HIV-1 [46–48], *Chlamydia* [49,50], *Diphtheria* [51–54], and *Cholera* [55–58]). An emerging concept is that the disulfide bonds function as a dynamic switch (breaking, forming and exchanging) to present proteins with different conformational and functional states at the cell surface [59,60], which is subjected to cellular redox regulation. The knowledge gained from this study not only has opened a new way for anthrax inhibition, but also can be applied to other related pathogens or toxins and to their interaction with host receptors.

## Supporting Information

S1 Fig Mass spectrometry analysis of the disulfide bond linkage of R318. (PDF)

S2 Fig Surface plasmon resonance (SPR) analysis of PA-receptor binding affinity. (PDF)

S3 Fig Kinetics of NBD emission. (PDF)
