# Supplementary material for: The Disulfide Bond Cys255-Cys279 in the Immunoglobulin-Like Domain of Anthrax Toxin Receptor 2 Is Required for Membrane Insertion of Anthrax Protective Antigen Pore
Source: PLoS One. 2015 Jun 24;10(6):e0130832. doi: 10.1371/journal.pone.0130832 (PMC4479931; doi:10.1371/journal.pone.0130832)
Supplement: S1 Fig — (PDF) [file pone.0130832.s001.pdf]

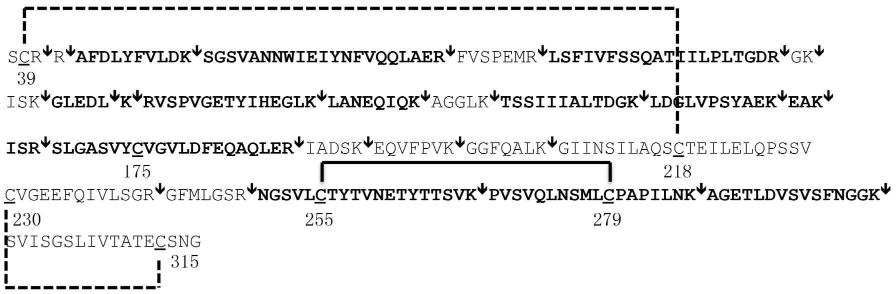

S1 Figure: Mass spectrometry analysis of the disulfide bond linkage of R318. The purified R318 was digested by trypsin and chymotrypsin (cutting sites labeled with arrows), and the digested peptides were subjected to LC/MS/MS analysis. ~55% peptides were recovered in LC/MS/MS (shown in bold letter). All of the Cys residues are underlined. The peptides containing C255 and C279 were detected to carry a disulfide bond. The peptide containing C175 was also detected, confirming that C175 is a free Cys residue as described in our early report (26). However, we could not detect the three peptides containing C39, (C218, C230) and C315. C39 and C218 reportedly form a disulfide bond in the VWA domain (14-16), while C230 and C315 are predicted to form another disulfide bond in the Ig domain. Since C218 and C230 are located in the same peptide after cleavage, it is very possible that the three peptides are linked by the disulfide bonds C39-C218 and C230-C315, which make LC/MS detection difficult.
