# Supplementary material for: The Disulfide Bond Cys255-Cys279 in the Immunoglobulin-Like Domain of Anthrax Toxin Receptor 2 Is Required for Membrane Insertion of Anthrax Protective Antigen Pore
Source: PLoS One. 2015 Jun 24;10(6):e0130832. doi: 10.1371/journal.pone.0130832 (PMC4479931; doi:10.1371/journal.pone.0130832)
Supplement: S2 Fig — (PDF) [file pone.0130832.s002.pdf]

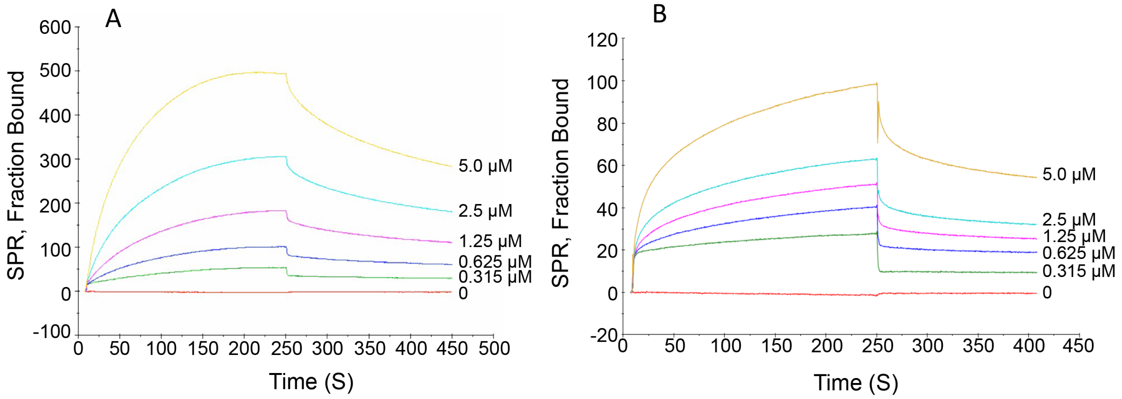

S2 Figure: **Surface plasmon resonance (SPR) analysis of PA-receptor binding affinity.** As indicated in an earlier report (14), the ultraslow dissociation kinetics made accurate measurement of the binding affinity between receptor and PA difficult using SPR. Here, using the TF-tagged receptors, TF-R318(WT) and TF-R318(C255/279A), we were able to measure the binding affinity between the TF-tagged receptors and PA_{83}. Briefly, PA_{83} was covalently coupled to a CM5 chip in a Biacore 100 system at 25˚C. 2μM of the protein was immobilized in sodium acetate pH 4.0 buffer at 5μl min^{-1} until the baseline reached <4,000 response units and then blocked with ethanolamine. Purified proteins of TF-R318(WT) (**A**) or TF-R318(C255/279A) (**B**) were diluted into HBS buffer (10mM Hepes pH 7.4, 150mM NaCl, 1 mM $MgCl_2$) and serial injections were made at a constant flow rate of 10μl min^{-1}. Concentrations of receptor proteins ranged from 38nM to 5μM. The CM5 chip was regenerated using 0.5 M $NaCO_3$ (pH 10.5) with a 30μl pulse, resulting in <1% loss of base line per injection. Kinetic and affinity studies were done on a 1:1 fit model and steady state, respectively.

The binding kinetics and affinity are shown in **S1 Table** below:

Binding Kinetics and Affinity between TF-R318 receptors and PA_{83}

	k_a (1/Ms)	k_d (1/s)	K_D (M)	Affinity K_D (M)
TF-R318(WT): PA_{83}	1222	0.001678	1.37E-06	5.91E-06
TF-R318(C255/279A):PA_{83}	8075	7.71E-04	9.55E-08	9.24E-07

As indicated in **S1 Table**, compared to TF-R318(WT), TF-R318(C255/279A) has a slightly higher affinity to PA_{83}, indicating that deletion of the disulfide bond C255-C279 does not reduce the binding affinity to PA_{83}.
