# Supplementary material for: The Disulfide Bond Cys255-Cys279 in the Immunoglobulin-Like Domain of Anthrax Toxin Receptor 2 Is Required for Membrane Insertion of Anthrax Protective Antigen Pore
Source: PLoS One. 2015 Jun 24;10(6):e0130832. doi: 10.1371/journal.pone.0130832 (PMC4479931; doi:10.1371/journal.pone.0130832)
Supplement: S3 Fig — (PDF) [file pone.0130832.s003.pdf]

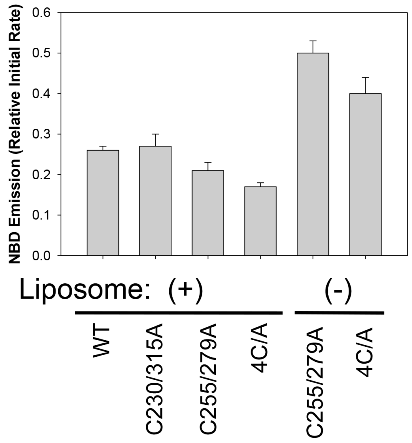


**S3 Figure: Kinetics of NBD emission.** Rates of NBD emission in **Fig 4** were calculated in SigmaPlot by fitting the curves to the single exponential equation f = a*[1 - exp(- b*x)], in which b is the rate of NBD emission at 544 nm.
